# Triple-catheter adjunctive technique through a 6-Fr distal access catheter for coil embolisation of splenic aneurysm: one balloon and two embolisation catheters via single access

**DOI:** 10.1093/bjrcr/uaag014

**Published:** 2026-04-11

**Authors:** Hiroshi Kuwamura, Yohsuke Suyama, Yasuhiro Enjoji, Ippei Ozaki, Yusuke Yamamoto, Mikiya Takao, Yoji Kishi, Hiroshi Shinmoto

**Affiliations:** Department of Radiology, National Defense Medical College, Tokorozawa, Saitama, 359-8513, Japan; Department of Radiology, National Defense Medical College, Tokorozawa, Saitama, 359-8513, Japan; Department of Radiology, National Defense Medical College, Tokorozawa, Saitama, 359-8513, Japan; Department of Radiology, National Defense Medical College, Tokorozawa, Saitama, 359-8513, Japan; Department of Radiology, National Defense Medical College, Tokorozawa, Saitama, 359-8513, Japan; Department of Hepato-Biliary-Pancreatic Surgery, National Defense Medical College, Tokorozawa, Saitama, 359-8513, Japan; Department of Hepato-Biliary-Pancreatic Surgery, National Defense Medical College, Tokorozawa, Saitama, 359-8513, Japan; Department of Radiology, National Defense Medical College, Tokorozawa, Saitama, 359-8513, Japan

**Keywords:** Splenic aneurysm, coil embolisation, balloon-assisted technique, double-catheter technique, distal access catheter

## Abstract

We present a novel triple-catheter adjunctive technique through a 6-Fr distal access catheter (DAC) for coil embolisation of an 18 mm wide-necked splenic aneurysm located at the bifurcation of the superior and inferior branches in a 63-year-old female. Despite the significantly tortuous splenic artery, the DAC was successfully navigated near the aneurysm, providing catheter stability. One 2.8-Fr balloon catheter and 2 2.2-Fr microcatheters were deployed simultaneously through the DAC to perform the balloon-assisted double-catheter technique. Dense packing was achieved with a volume embolisation ratio of 39.2%, while preserving the superior branch flow. Post-embolisation angiography revealed complete aneurysmal occlusion without splenic infarction. This technique presents a viable solution for complex visceral aneurysms in tortuous anatomies, enabling dense packing while preserving the parent artery, without requiring multiple access sites or larger guiding catheters.

## Background

Splenic aneurysms, the most common type of visceral artery aneurysms, have a high mortality rate if ruptured.^1^ Endovascular techniques, such as branch isolation and aneurysm packing, offer treatment options. Although branch isolation effectively occludes aneurysms, it significantly increases the risk of splenic ischaemia.^2^^,^^3^ Aneurysmal packing prioritises the preservation of splenic blood flow. Packing density is objectively evaluated using the volume embolisation ratio (VER), calculated as follows: VER = (total volume of deployed coils/aneurysm volume) × 100. To prevent aneurysm recurrence, achieving a VER of at least 24% is crucial, emphasising the importance of dense packing.^4^

The combination of balloon-assisted and double-catheter techniques allows for both blood flow preservation and dense aneurysm packing.^5^^,^^6^ However, this technique typically requires multiple arterial access sites or a larger-diameter guiding catheter. The tortuosity of the splenic artery poses challenges to catheter navigation and stability, necessitating the use of a distal access catheter (DAC) to enhance procedural success.^7^

In this report, we present a case of a splenic aneurysm located at a bifurcation with a tortuous access route. We successfully performed tight packing of the aneurysm using the combination of balloon-assisted and double-catheter techniques through a single 6-Fr DAC.

## Clinical presentation

Written informed consent was obtained from the patient for publication of this case report, including the accompanying images. Institutional review board approval was waived for this case report. A 63-year-old female was referred to our hospital for endovascular treatment of a splenic aneurysm detected during a routine health check-up at another facility. The patient was asymptomatic and had no significant past medical history or family history of aneurysms or other vascular complications.

**Figure 1:**
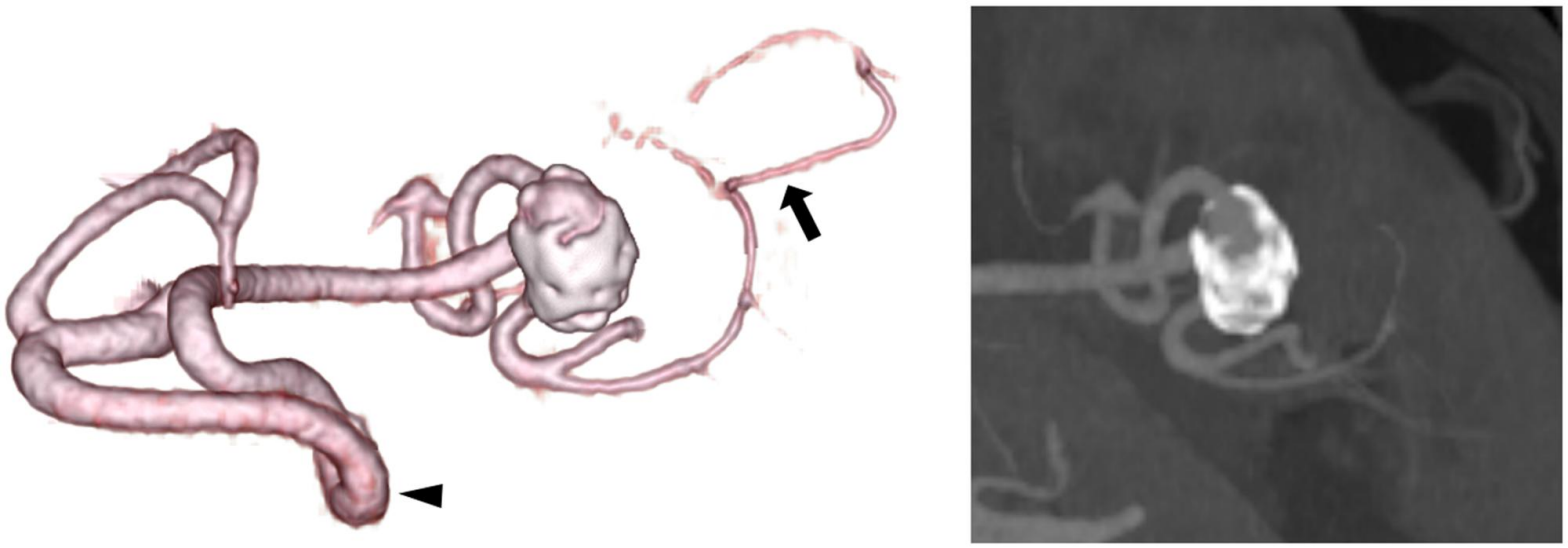
Contrast-enhanced computed tomography (CECT) before endovascular treatment. The volume rendering and maximum intensity projection reconstruction images show a wide-necked 18 × 10 × 10 mm splenic aneurysm. The mid-portion of the splenic artery exhibits significant tortuosity and angulation (black arrow head). The aneurysm is located at the bifurcation of the superior and inferior branches of the splenic artery. The inferior branch is connected to the left gastroepiploic artery (black arrow).

Contrast-enhanced computed tomography (CECT) revealed a wide-necked splenic aneurysm measuring 18 × 10 × 10 mm (Figure 1). The aneurysm was located at the bifurcation of the superior and inferior branches of the splenic artery. The superior branch of the splenic artery lacked visible collateral circulation, indicating a high risk of splenic ischaemia if embolised. Conversely, the inferior branch was connected to the left gastroepiploic artery, suggesting a lower risk of ischaemic complications caused by embolisation. The splenic artery had a tortuous segment with significant curvature and angulation, indicating potential technical difficulties in endovascular access.

## Endovascular treatment procedure

**Figure 2:**
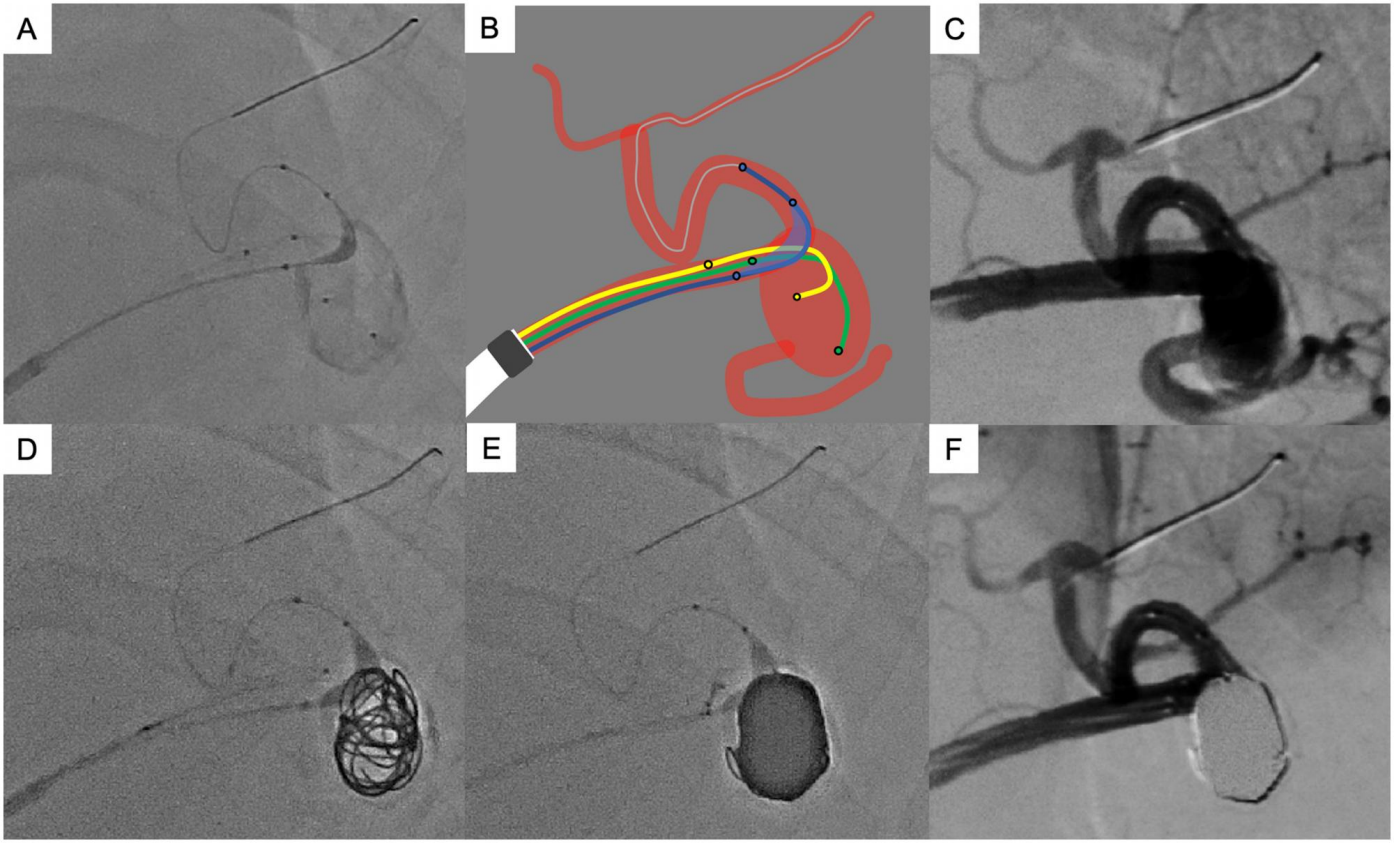
Endovascular treatment of splenic artery aneurysm using triple-catheter technique through a 6-Fr distal access catheter (DAC). (A) Fluoroscopic image showing 3 microcatheters deployed through a 6-Fr DAC into the splenic aneurysm. A balloon catheter is positioned across the neck of the aneurysm. (B) Schematic illustration of triple-catheter configuration through a 6-Fr DAC. The DAC (white) is advanced to the proximal aspect of the aneurysm. One balloon catheter (blue) is positioned across the neck of the aneurysm, whereas 2 embolisation microcatheters (yellow and green) are deployed simultaneously within the aneurysm sac. (C) Splenic angiogram through the 6-Fr DAC with all 3 catheters in place demonstrates an 18mm splenic aneurysm at the bifurcation of the superior and inferior branches with excellent contrast agent injection ability. (D) Fluoroscopic image after deployment of the 2 framing coils. The balloon preserves the superior branch of the splenic artery while facilitating the creation of a robust coil frame. (E) Fluoroscopic image after balloon-assisted and double-catheter coil embolisation with 15 coils. Dense intra-aneurysmal packing is achieved with a volume embolisation ratio (VER) of 39.2%. (F) Post-embolisation angiogram of the splenic artery shows complete aneurysm occlusion with preservation of flow in the superior branch.

Figure2 shows the details of coil embolisation. The procedure was performed under local anaesthesia via the right femoral artery. A 6-Fr shepherd hook-shaped guiding sheath (Parent Plus 60 SHC; Medikit, Tokyo, Japan) was inserted into the celiac artery. Celiac angiography revealed a splenic aneurysm located at the bifurcation of the superior and inferior branches, with significant tortuosity in the mid-portion of the splenic artery, presenting a substantial challenge for catheter navigation. Following the placement of the guiding sheath, systemic heparinisation was achieved with an intravenous bolus of 3000 U heparin.

A 6-Fr DAC (Cerulean DD6; Medikit, Tokyo, Japan) was navigated to the proximal aspect of the splenic aneurysm using an inner catheter (4.2-Fr Fubuki; Asahi Intecc, Tokyo, Japan) and a guidewire (0.035-inch Radifocus; Terumo, Tokyo, Japan) to overcome the tortuous anatomy. A balloon catheter (4 mm × 20 mm Scepter C; Terumo, Tokyo, Japan) and 2 microcatheters (Phenom 17; Medtronic, Minnesota, USA) were inserted to perform balloon-assisted and double-catheter embolisation through the DAC. The balloon catheter was positioned across the aneurysm neck in the superior branch to preserve the blood flow. Although an optimal barrel view was not achievable due to C-arm movement limitations, the best possible working angle was obtained.

Two framing coils were used to create a stable scaffold frame: (1) 14 mm × 47 cm MICRUSFRAME S (Johnson and Johnson, New Jersey, USA) and (2) 12 mm × 40 cm MICRUSFRAME S. Subsequently, multiple filling and finishing coils with decreasing primary and secondary coil diameters were sequentially deployed through the 2 microcatheters. The coils used for filling and finishing included: (3) 12 mm × 45 cm Target XL (Stryker, Michigan, USA), (4) 10 mm × 40 cm Target XL, (5) 10 mm × 40 cm Target XL, (6) 8 mm × 30 cm Target XL, (7) 7 mm × 21 cm GALAXY G3 (Johnson and Johnson, New Jersey, USA), (8) 7 mm × 21 cm GALAXY G3, (9) 6 mm × 20 cm Avenir Helical Filling (Wallaby, China), (10) 5 mm × 20 cm Avenir Helical Filling, (11) 5 mm × 20 cm Avenir Helical Filling, (12) 5 mm × 20 cm Avenir Helical Filling, (13) 5 mm × 20 cm Avenir Helical Filling, (14) 4 mm × 20 cm Avenir Helical Filling, and (15) 4 mm × 20 cm Avenir Helical Filling. The aneurysm volume was calculated as 942 mm³ based on 18 × 10 × 10 mm diameters, and the total volume of the 15 coils was 369 mm³, resulting in a packing density VER of 39.2%.

**Figure 3:**
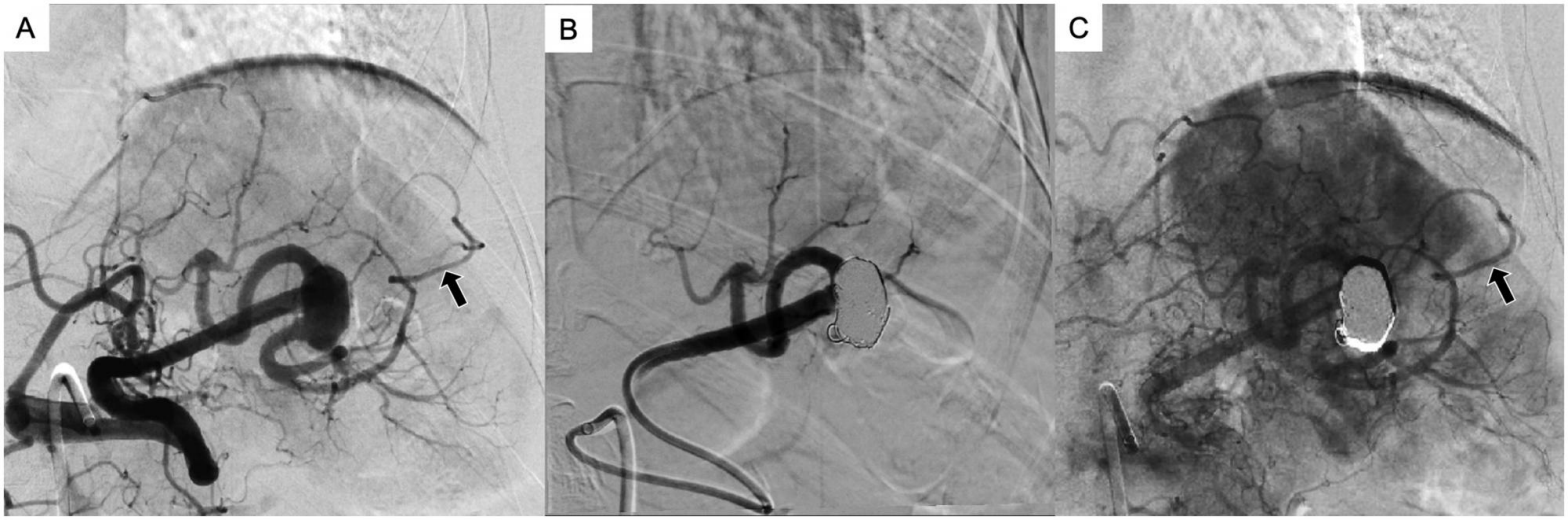
Comparison of pre-/post-embolisation celiac and splenic angiography. (A) Pre-embolisation celiac angiography shows the left gastroepiploic artery (black arrow) continuous with the inferior branch of the splenic artery. (B) Post-embolisation selective splenic angiography demonstrates preservation of the superior branch, whereas the inferior branch is no longer visualized. (C) Post-embolisation celiac angiography reveals the inferior branch of the splenic artery opacified via collateral circulation from the left gastroepiploic artery (black arrow).

Post-embolisation splenic angiography confirmed complete occlusion of the aneurysm while preserving the blood flow in the superior branch. Celiac angiography demonstrated patency of the inferior branch of the splenic artery through collaterals from the left gastroepiploic artery, with no evidence of splenic infarction (Figure 3). The procedure was completed without complications, and haemostasis was achieved by manual compression after sheath removal.

## Follow up after coil embolisation

**Figure 4:**
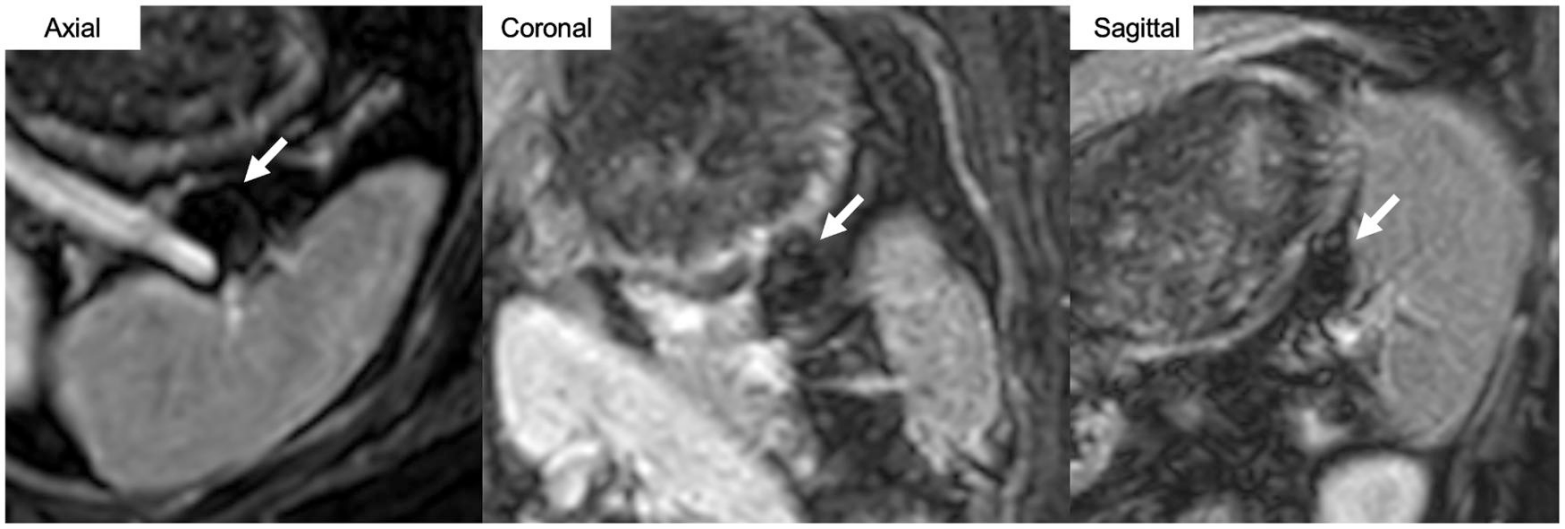
Contrast-enhanced magnetic resonance image after splenic aneurysm embolisation. Contrast-enhanced magnetic resonance imaging performed 2 months after coil embolisation shows complete occlusion of the splenic aneurysm with no evidence of coil compaction, aneurysm recurrence, and splenic infarction.

Contrast-enhanced magnetic resonance imaging (CE-MRI) performed 2 months after coil embolisation demonstrated complete occlusion of the splenic aneurysm, with no evidence of coil compaction or aneurysm recurrence (Figure 4). CE-MRI was selected instead of CECT because it produces fewer coil-related artefacts and allows clearer evaluation of contrast enhancement within the coil mass. No splenic infarctions or other procedure-related complications were observed. The patient was followed up in our hospital for 3 months after coil embolisation, during which no recurrences or adverse events occurred.

## Discussion

We successfully performed dense packing (VER: 39.2%) of a splenic artery aneurysm using a combination of the balloon-assisted and double-catheter techniques through a single DAC, which was useful for overcoming the significant tortuosity of the splenic artery. This approach is especially effective for aneurysms located at bifurcations with tortuous access routes.

Ruptured splenic aneurysms are associated with high mortality and are usually considered for treatment when their size exceeds approximately 20 mm.^1^ Coil embolisation strategies for splenic aneurysms include aneurysmal packing and branch isolation.^3^ Aneurysmal packing is less likely to cause ischaemic complications in the spleen; however, this technique carries the risk of coil compaction and aneurysm recurrence when dense packing cannot be achieved.^4^ Conversely, branch isolation offers a more definitive embolisation method but increases the risk of splenic infarction.^2^^,^^3^

In this case, we opted to treat the 18 mm aneurysm, which was slightly below the 20 mm threshold, using aneurysmal packing. This decision was influenced by the patient’s preference for treatment over observation and the desire to preserve the splenic artery blood flow. Previous reports have indicated that isolation of splenic aneurysms may lead to splenic infarction,^2^^,^^3^ highlighting the importance of preserving splenic blood flow during embolisation. The splenic inferior branch was connected distally to the left gastroepiploic artery, which served as a collateral pathway, allowing the preservation of only the superior branch. Yasumoto et al. reported a significant difference in mean packing density between small (<20 mm; VER 22%) and large (≥20 mm; VER 15%) aneurysms (*P* = 0.0045), and no compaction or recanalization occurred in aneurysms with a VER of at least 24%.^4^ Smaller aneurysms (<20 mm) tend to achieve a higher VER during packing embolisation than larger aneurysms (>20 mm), thus reducing the recurrence risk and requiring fewer coils. Larger aneurysms often require branch isolation because achieving high-density packing is difficult and using numerous coils is expensive. Therefore, we performed dense packing for an 18 mm splenic aneurysm while preserving the splenic superior branch flow.

Various adjunctive techniques for aneurysmal packing can be employed to enhance VER and preserve the neck of the aneurysm.^1^ The balloon-assisted technique is particularly effective for preserving branch vessels without the need for antiplatelet therapy. The double-catheter technique is beneficial for creating a robust frame in irregularly shaped aneurysms and ensuring tight packing.^8^ Although stent-assisted techniques offer improved branch preservation, they require antiplatelet therapy due to the use of neck-bridging stents.

Multiple puncture access sites or a larger guiding catheter are necessary to perform the combined balloon-assisted and double-catheter techniques.^5^^,^^6^ The first method involves dual access via the bilateral femoral arteries, necessitating 2 puncture sites and consequently increasing invasiveness. The second method employs a larger-diameter guiding catheter, such as a 7-Fr guiding catheter or 6-Fr guiding sheath (equivalent to an 8-Fr outer diameter). However, navigating these larger sheaths through tortuous vessels presents significant challenges.

**Figure 5:**
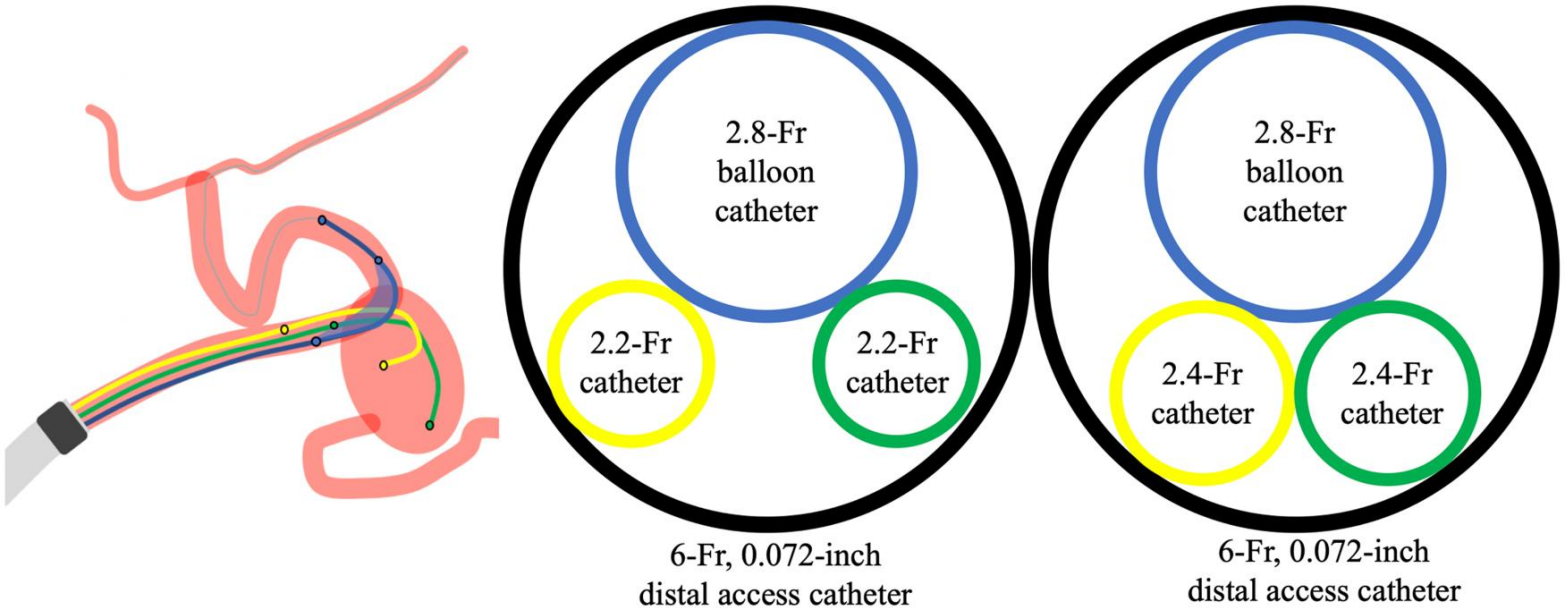
Illustration of triple microdevices compatibility and feasibility within a 6-Fr 0.072-inch distal access catheter (DAC). The configuration of 1 2.8-Fr balloon catheter and 2 2.2-Fr catheters allows for sufficient lumen patency for contrast agent injection without catheter interference through a 6-Fr 0.072-inch DAC. Conversely, the combination of 1 2.8-Fr balloon and 2 2.4-Fr catheters results in inadequate space, which impedes catheter insertion, manoeuvrability, and contrast medium injection.

In our case, a combination of balloon-assisted and double-catheter techniques using a single 6-Fr DAC was chosen to preserve the superior branch of the splenic artery for tight packing of the aneurysm. A 6-Fr shepherd hook-shaped guiding sheath was strategically placed in the celiac artery to ensure catheter stability during embolisation. Through this sheath, a 6-Fr DAC was navigated beyond the tortuous vessels near the splenic aneurysm, enabling stable coil embolisation and contrast injection. Typically, a 6-Fr guiding catheter can accommodate both a 2.8-Fr balloon and a 2.4-Fr embolisation catheter; ^9^^,^^10^ however, a third 2.4-Fr catheter is not feasible. Even if a third catheter is inserted, catheter interference may impede the operation and hinder contrast injection from the guiding catheter. To overcome these technical limitations, we utilised the Cerulean DD6, which features a slightly larger inner lumen of 0.072-inch, alongside the Phenom 17, which has a proximal outer diameter of 2.2-Fr, slightly smaller than that of other microcatheters. This configuration enabled the deployment of triple microdevices through a single 6-Fr 0.072-inch DAC, incorporating 1 2.8-Fr balloon and 2 2.2-Fr microcatheters (Figure 5 and Table 1). In this setup, inserting the balloon catheter first is crucial because its balloon portion, being the widest part, would interfere with the other microcatheters if inserted later. This triple-catheter setup facilitates tight packing and balloon neck remodelling while maintaining catheter stability and the ability to inject contrast materials, thereby verifying neck preservation.

**Table 1:** Compatibility chart of 6-Fr 0.072-inch distal access catheter and microcatheters.

First catheter	Second catheter	Third catheter	Catheters insertion	Contrast agent injection
2.8-Fr balloon	2.4-Fr catheter	N/A	possible	possible
2.8-Fr balloon	2.6-Fr catheter	N/A	possible	possible
2.8-Fr balloon	2.8-Fr balloon	N/A	impossible	N/A
2.8-Fr balloon	2.2-Fr catheter	2.2-Fr catheter	possible^a^	possible
2.8-Fr balloon	2.4-Fr catheter	2.4-Fr catheter	impossible^b^	impossible^b^
2.8-Fr balloon	2.6-Fr catheter	2.6-Fr catheter	impossible	N/A

The Fr (French) size of a catheter represents its proximal outer diameter.

N/A, Not Applicable.

a Inserting the balloon catheter first is essential due to its larger balloon portion than the catheter shaft, which interferes with other catheters if inserted later.

b This combination of 3 catheters is usually impossible to insert. Even when insertion is achieved, severe catheter interference significantly impedes catheter manoeuvrability and contrast agent injection.

A notable limitation of this approach is the inability to deploy high-volume coils with larger primary diameters, such as 17-inch, 20-inch, and 35-inch coils, using Phenom 17, which may be desirable for larger aneurysms. Additionally, this setup does not allow for the combination of 2 balloons and 1 embolisation catheter, which might be preferable in certain complex cases. This report is based on a single case, and remains unclear whether the approach effectively enhances VER and reduces coil compaction and recurrence rates. Future studies with a larger number of cases are needed to understand these potential benefits better.

In conclusion, we successfully performed dense packing (VER 39.2%) of splenic aneurysms using a combination of balloon-assisted and double-catheter techniques through a single access route, utilising a 6-Fr 0.072-inch DAC to simultaneously insert 3 microdevices (1 2.8 Fr-balloon and 2 2.2 Fr-embolisation catheters). This method was deemed useful for aneurysms located in tortuous access routes and bifurcations, where branch blood flow preservation is necessary.

## Informed consent statement

The patient provided written informed consent for the publication of this case report, including the accompanying images. The need for institutional review board approval was waived for this case report.

## Learning points

- Distal access catheters effectively stabilise catheter systems when navigating highly tortuous access routes to aneurysms.
- The 6-Fr 0.072-inch distal access catheter can accommodate the simultaneous insertion of a 2.8-Fr balloon catheter and 2 2.2-Fr microcatheters, enabling a triple-catheter adjunctive technique.
- The combination of balloon-assisted and double-catheter techniques allows for dense coil packing while preserving the parent artery.
